# Neuroanatomical and Functional Correlates in Depressive Spectrum: A Narrative Review

**DOI:** 10.3390/jpm15100478

**Published:** 2025-10-02

**Authors:** Giulio Perrotta, Anna Sara Liberati, Stefano Eleuteri

**Affiliations:** 1 Department of Human and Social Sciences, Universitas Mercatorum, Piazza Mattei 10, 00186 Rome, Italy; stefano.eleuteri@unimercatorum.it; 2 Institute for the Study of Psychotherapies (ISP), Via San Martino Della Battaglia 31, 00185 Rome, Italy; 3 Faculty of Psychology, Università Telematica Internazionale “Uninettuno”, 00186 Rome, Italy; annasaraliberati@gmail.com

**Keywords:** depressive spectrum, neuroanatomical correlates, prefrontal cortex, anterior cingulate cortex, amygdala, hippocampus, default mode network, major depressive, melancholic depression, atypical depression, dysthymia, neuroimaging, biotypes

## Abstract

Depressive spectrum disorders are considered among the most common in the general population. Major depressive disorder and persistent depressive disorder (or dysthymia) are the most recognized, but other depressive disorders exist with varying or no specificity. The main difference between major depressive disorder and dysthymia lies in the duration and intensity of symptoms. Improving our understanding of its etiology and pathogenesis must be a priority for health and safety. Given the complexity of the evidence in the literature, it was deemed useful to provide a comprehensive summary of the neuroanatomical dysfunctions currently identified, with particular attention to the anterior and medial cingulate cortex, dorsolateral and ventromedial prefrontal cortex, posterior parietal cortex, insula, amygdala, and hippocampus. Significant neural network alterations include hyperconnectivity of the default mode network (DMN), impairment of the executive control network (ECN), and dysfunction of the salience network (Salience Network). Neurophysiological markers reveal frontal alpha asymmetries and front-striatal metabolic alterations. Studying neural correlates is essential to deepen our understanding of the depressive spectrum and the development of personalized therapeutic interventions, including noninvasive neurostimulation techniques and target-specific pharmacological therapies, opening new avenues for translational research in neuropsychiatric settings.

## 1. Introduction and Research Objective

Depressive spectrum (DS) stands as one of the most prevalent psychiatric conditions worldwide, affecting an estimated 10–15% of the global population during lifetime [1]. Beyond its statistical prominence, DS represents the leading cause of disability in developed nations, generating substantial socio-economic burdens through direct healthcare costs, lost productivity, and reduced quality of life [2]. The clinical presentation of the disorder includes a complex symptomatology that extends well beyond mood disorders (characteristic of the depressive spectrum), including cognitive deficits, neurovegetative changes, and profound alterations in psychosocial functioning [3]. The phenomenological complexity of depression reflects its multifactorial aetiology, where genetic predisposition, neurobiological vulnerabilities, psychological factors, and environmental stressors converge in intricate patterns of interaction [4]. This complexity has long challenged researchers and clinicians alike, driving the need for a deeper understanding of the biological substrates that underlie this devastating condition. Understanding the neural correlates of depression has emerged as a critical endeavour for several compelling reasons. First, identifying specific neurobiological alterations provides essential insights into the pathophysiological mechanisms that drive depressive symptoms, moving beyond descriptive phenomenology toward mechanistic understanding [5]. Second, neural markers offer potential avenues for improving diagnostic precision, particularly valuable when facing atypical presentations or complex comorbidities that complicate traditional diagnostic approaches [6]. Perhaps most importantly, knowledge of dysfunctional neural circuits guides the development of innovative therapeutic strategies, including targeted neurostimulation techniques and personalized pharmacological interventions [7]. Contemporary neuroimaging research has revealed a consistent pattern of alterations in depression, primarily affecting the dorsolateral and ventromedial prefrontal cortex, accompanied by amygdala hyperactivation, hippocampal volumetric reductions, and anterior cingulate cortex dysfunction [8,9,10]. Equally significant are the alterations in large-scale neural networks, including hyperconnectivity of the default mode network, impairment of the executive control network, and dysfunction of the salience network [11]. Neurophysiological studies have further identified frontal alpha asymmetries and frontostriatal metabolic alterations that correlate with symptom severity and treatment response [12]. Recent advances in computational psychiatry and machine learning approaches have revealed the existence of distinct neurobiological biotypes. These findings suggest that different depressive presentations (including melancholic, atypical, and chronic forms) may represent distinct neurobiological entities rather than simple phenotypic variations of a single disorder. This paradigm shift toward biologically informed subtyping holds profound implications for precision medicine approaches in psychiatry [13,14]. The identification of these neural correlates holds profound implications for advancing our understanding of DS and developing more effective, personalized therapeutic approaches. This knowledge opens new possibilities for noninvasive neurostimulation techniques and target-specific pharmacological therapies, while establishing a foundation for translational research in neuropsychiatric settings [15]. In clinical practice, individual depressive symptoms are often confused with structured or complex disorders, leading to a misinterpretation that can lead psychiatrists to prescribe psychotropic medications, relegating psychotherapy to mere support or minimizing its importance, thus risking chronicizing the patient’s symptoms. To reduce this risk, the “Perrotta Depressive Symptoms Assessment” (PDSYA) was recently published for differential diagnosis in disorders with depressive symptoms. In the pilot study conducted, statistical analyses validating the model demonstrated its validity, emphasizing the analysis of all specific depressive traits to reduce diagnostic errors [16]. Therefore, this narrative review aims to provide a comprehensive synthesis of current scientific evidence regarding the neural correlates of DS. Through this contribution, we seek to clarify what we know about the depressed brain but also how this knowledge can be translated into better outcomes for individuals suffering from this debilitating condition. The narrative and descriptive nature of the contribution does not reduce the importance of the results identified in this work, which, however, does not present a meta-analytic or systematic structure, and this could be considered a limitation of the results presented, to be taken into consideration in relation to the conclusions.

## 2. Theoretical Models and General Neurobiology

### 2.1. Monoaminergic Model

The monoaminergic theory remains fundamental to our understanding of DS, proposing that dysregulation of serotonergic, noradrenergic, and dopaminergic neurotransmitter systems underlies its complex symptomatology. While initially conceptualized as simple neurotransmitter depletion, contemporary research has revealed far more nuanced alterations involving receptor sensitivity, transporter efficiency, and downstream signalling cascades [17]. Serotonergic dysfunction in depression extends beyond the dorsal raphe nucleus to encompass widespread alterations in cortical and limbic projections, particularly affecting mood regulation and emotional processing circuits. Research demonstrates the presence of altered serotonergic metabolism, impaired synaptic availability, and disturbed receptor functionality in key brain regions. The complexity of serotonergic alterations helps explain both the heterogeneity of depressive presentations and the variable responses to serotonergic interventions [18]. The noradrenergic system, primarily originating from the locus coeruleus, shows significant dysfunctions that manifest clinically through specific features, including concentration difficulties, sleep disturbances, and altered psychomotor activity. These alterations profoundly impact arousal regulation, attentional mechanisms, and stress response modulation, thereby contributing to the cognitive and somatic symptoms that often dominate the clinical picture in DS [19]. Finally, dopaminergic dysfunction—particularly evident in the anhedonic components of depression—reflects specific alterations in reward processing circuits that include the ventral tegmental area and the nucleus accumbens. Impairment of this system leads to decreased motivation and pleasure-seeking behaviours that characterize central aspects of the depressive experience. Recognition of dopaminergic involvement therefore has important therapeutic implications, particularly useful for better understanding the clinical characteristics and aetiology of treatment-resistant DS [20].

### 2.2. Neuroinflammation and Oxidative Stress

The emergence of neuroinflammation as a key mechanism in DS represents a paradigmatic shift toward understanding the disorder itself as a systemic condition with significant immune system involvement. This perspective has opened entirely new avenues for both understanding and treating DS [21]. Microglial activation, triggered by diverse stressors including psychosocial adversity, infection, or metabolic dysfunction, initiates a cascade of inflammatory processes that directly interfere with normal neurotransmission. Elevated pro-inflammatory cytokines, including interleukin-1β (IL-1β), tumor necrosis factor-α (TNF-α), and interleukin-6 (IL-6), activate the indoleamine 2,3-dioxygenase pathway, leading to tryptophan degradation and consequent serotonin depletion while simultaneously promoting the production of neurotoxic kynurenine metabolites [22]. Beyond neurotransmitter effects, chronic neuroinflammation contributes to hippocampal neurogenesis suppression and increased oxidative stress burden, creating a self-perpetuating cycle of cellular damage that compromises synaptic integrity and neural plasticity mechanisms. This inflammatory framework provides compelling explanations for the bidirectional relationships between DS and various medical comorbidities, including cardiovascular disease, diabetes, and autoimmune conditions, while offering novel therapeutic targets for intervention [23].

### 2.3. Neuroplasticity and Neurotrophic Factors (BDNFs)

Brain-derived neurotrophic factor (BDNF) has emerged as a critical mediator of synaptic plasticity, adult neurogenesis, and neural adaptation processes that are fundamentally compromised in depression and mood disorders [24]. The consistent finding of reduced BDNF levels in DS patients, particularly in hippocampal and prefrontal cortical regions, represents more than a simple biomarker; it actively contributes to pathophysiology by compromising synaptic repair mechanisms, dendritic branching, and neural adaptation processes essential for emotional regulation and cognitive flexibility [25]. The BDNF deficit hypothesis has proven particularly valuable for understanding treatment mechanisms across diverse therapeutic modalities. Effective interventions, whether pharmacological, psychological, or neurostimulation-based, consistently demonstrate the capacity to restore BDNF levels and promote neuroplastic recovery. This convergent mechanism suggests that therapeutic efficacy may fundamentally depend on the restoration of adaptive neuroplasticity, providing a unifying framework for understanding how disparate treatments achieve clinical benefit in DS [26].

## 3. Primarily Involved Neural Circuits and Brain Network Alterations

**Figure 1:**
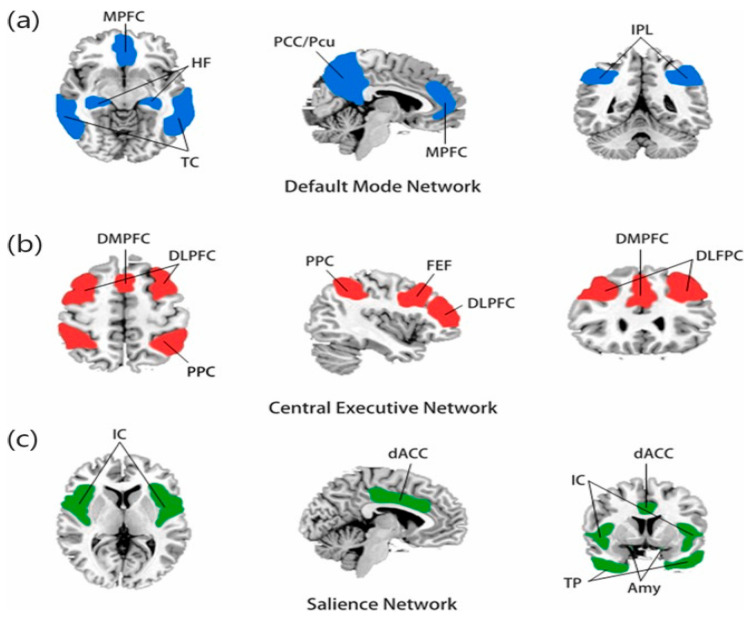
(**a**) The default mode network is mainly composed of the medial prefrontal cortex (MPFC) and posterior cingulate cortex/precuneus (PCC/PCu), and the temporal cortex (TC), hippocampus formation (HF), and inferior parietal lobule (IPL) are also closely related to this network. (**b**) The central executive network (CEN) is mainly composed of the dorsolateral prefrontal cortex (DLPFC) and posterior parietal cortex (PPC), the dorsomedial prefrontal cortex (DMPFC), and the frontal eye field (FEF). (**c**) The salience network is composed of the insular cortex (IC), dorsal anterior cingulate cortex (dACC), temporal pole (TP), and amygdala (Amy) [27].

The network-neuroscience approach has fundamentally transformed our understanding of DS by shifting focus from isolated brain regions to dynamic interactions between specialized neural networks. This paradigmatic shift has revealed that DS emerges from disrupted functional balance between three primary networks (Figure 1), each contributing distinct pathophysiological mechanisms to the disorder’s complex presentation [11].

### 3.1. Default Mode Network (DMN)

The default mode network (DMN), encompassing the medial prefrontal cortex, posterior cingulate cortex, and angular gyrus, normally exhibits high activity during rest and introspective states. In DS, however, this network demonstrates pathological hyperconnectivity that correlates directly with the intensity of ruminative phenomena [28]. This aberrant hyperactivation creates a neurobiological substrate for the persistent, self-referential negative thinking patterns that characterize depressive cognition. The DMN’s hyperconnectivity in DS represents one of the most robust findings in neuroimaging research, consistently identified across diverse populations and methodological approaches. This network alteration provides a compelling neurobiological explanation for the subjective experience of being trapped in repetitive negative thoughts, while offering potential targets for therapeutic intervention through techniques designed to modulate network connectivity [29].

### 3.2. Executive Control Network (ECN)

The executive control network (ECN), comprising the dorsolateral prefrontal cortex and posterior parietal cortex, shows marked functional impairments in DS that manifest through reduced connectivity and compromised top-down emotional regulation. These network dysfunctions contribute directly to the cognitive symptoms of DS, including concentration problems, decision-making difficulties, and the inability to effectively modulate emotional responses to environmental challenges. The compromised cognitive control and reduced capacity for goal-directed behaviour observed in depressed patients directly reflect the functional limitations imposed by ECN dysfunction. This creates a cascade of cognitive impairments that significantly impact daily functioning and may influence therapeutic responsiveness, as effective engagement with psychological interventions often requires intact executive functioning [30].

### 3.3. Salience Network (SN)

The salience network (SN), anchored by the anterior insula and dorsal anterior cingulate cortex, demonstrates significant dysfunction in DS, particularly affecting the capacity for appropriate attentional orientation and relevant stimulus selection. This network presents specific dysfunctions in the ability to orient attention toward relevant external stimuli, determining compromised attentional switching and contributing to the persistence of dysfunctional internal attentional focus [31]. These alterations contribute to the characteristic cognitive biases that perpetuate depressive thinking patterns, as the impaired salience detection mechanisms fail to appropriately direct attention away from negative internal states toward potentially adaptive external stimuli. The disrupted balance between these networks—characterized by DMN hyperactivation, ECN hypoactivation, and SN dysfunction—creates a pathological state where individuals become trapped in repetitive negative thinking while simultaneously losing the cognitive flexibility necessary for adaptive emotional regulation and problem-solving. This network-level understanding has profound implications for therapeutic interventions, as effective treatments must address not only individual network dysfunctions but also restore the dynamic balance between these interconnected systems that collectively support healthy emotional and cognitive functioning [32].

## 4. DS: Clinical and Neurobiological Distinctions

The heterogeneity of DS has long been recognized clinically, with various attempts to identify meaningful subtypes based on symptom profiles, treatment response, and course of illness. Recent neuroimaging advances have provided unprecedented insights into the distinct neurobiological substrates underlying different depressive presentations [33,34]. This section examines the three major depressive subtypes with established neurobiological correlates: major depressive disorder (single episode and recurrent), melancholic depression, atypical depression, and persistent depressive disorder (dysthymia).

Major Depressive Disorder: core clinical and neurobiological features. DS, as defined by DSM-5-TR criteria [3], represents the most extensively studied form of depression. The neurobiological signature of classic DS encompasses the core alterations: prefrontal hypoactivation, amygdala hyperresponsivity, hippocampal volume reductions, and disrupted network connectivity patterns. Recent large-scale neuroimaging studies have identified robust biomarkers that distinguish DS patients from healthy controls with 70–85% accuracy. The most consistent findings include reduced functional connectivity within the ECN, increased DMN activity, and altered limbic-prefrontal connectivity patterns [35].

Melancholic depression: reward circuit dysfunction. Melancholic depression represents a severe subtype characterized by profound anhedonia, psychomotor changes, early morning awakening, marked appetite disturbance, and excessive guilt. This subtype affects approximately 25–30% of DS patients and demonstrates distinct neurobiological features that differentiate it from other depressive forms [36]. Clinical features encompass severe anhedonia and loss of reactivity to pleasurable stimuli, a distinct quality of depressed mood different from grief, significant psychomotor agitation or retardation, early morning awakening with mood worst in the morning, marked anorexia or weight loss, and excessive or inappropriate guilt. Neurobiological correlates reveal that melancholic depression shows particularly pronounced dysfunction in reward processing circuits, with significant alterations in dopaminergic pathways connecting the ventral tegmental area to the nucleus accumbens and prefrontal cortex [37]. Neuroimaging studies reveal striatal dysfunction with a 40–60% reduction in ventral striatum activation during reward anticipation tasks, correlating with anhedonia severity [38]. Dopaminergic alterations manifest through decreased dopamine transporter availability in the caudate nucleus and putamen, with 20–35% reductions compared to non-melancholic depression [39]. Anterior cingulate hyperactivity demonstrates pronounced hypermetabolism in the subgenual anterior cingulate cortex (sgACC), with glucose uptake increases of 35–50% compared to healthy controls [40]. HPA axis dysfunction presents with enlarged adrenal glands and elevated cortisol levels, correlating with hippocampal volume reductions of 15–25% [41].

Atypical depression: unique metabolic and connectivity patterns. Atypical depression, affecting 15–25% of DS patients, is characterized by mood reactivity, hypersomnia, hyperphagia, leaden paralysis, and interpersonal rejection sensitivity. Despite its name, atypical depression is relatively common and shows distinct neurobiological features [42]. Clinical features include mood reactivity with brightening in response to positive events, significant hypersomnia or hyperphagia, leaden paralysis manifesting as heavy feelings in limbs, interpersonal rejection sensitivity, and an earlier age of onset compared to melancholic depression. Recent neuroimaging studies have identified unique white matter integrity patterns in atypical depression, with distinct alterations compared to melancholic subtypes [43]. Metabolic differences show increased metabolic activity in the orbitofrontal cortex and decreased activity in the dorsolateral prefrontal cortex, opposite to patterns seen in melancholic depression [44]. Limbic hyperactivity presents through enhanced amygdala responsivity to emotional stimuli, particularly negative social cues, correlating with rejection sensitivity [45]. Hypothalamic dysfunction manifests through altered hypothalamic–pituitary–adrenal axis function with paradoxical patterns of cortisol secretion and inflammatory marker elevation. White matter alterations reveal reduced fractional anisotropy in the anterior limb of the internal capsule and corpus callosum, distinct from the posterior white matter changes seen in melancholic depression [46].

Persistent depressive disorder (Dysthymia): chronic structural alterations. Persistent depressive disorder (PDD), previously known as dysthymia, represents a chronic form of depression lasting at least two years in adults. While symptoms may be less severe than in major depressive episodes, the chronic nature of PDD produces distinct neurobiological adaptations [47]. Clinical features encompass chronic depressed mood for most days over at least two years and the presence of two or more symptoms, including appetite changes, sleep disturbances, fatigue, low self-esteem, concentration problems, and hopelessness. Patients are never symptom-free for more than two months during the two-year period and experience significant functional impairment despite seemingly “milder” symptoms. Neurobiological studies reveal that the chronic nature of PDD produces progressive structural and functional brain changes that differ from episodic DS [48]. Progressive volume loss demonstrates greater hippocampal and prefrontal cortical volume reductions compared to episodic DS, with 12–20% decreases correlating with illness duration [49]. White matter changes manifest through extensive hyperintensities and reduced connectivity between frontal and limbic regions, more pronounced than in DS [50]. Persistent network dysfunction exhibits chronic DMN hyperconnectivity that persists even during periods of relative symptom improvement [51]. Neurotrophic deficits show consistently reduced BDNF levels with impaired neuroplastic recovery, contributing to treatment resistance commonly observed in PDD [52].

## 5. Neural Correlates Involved in DS

**Figure 2:**
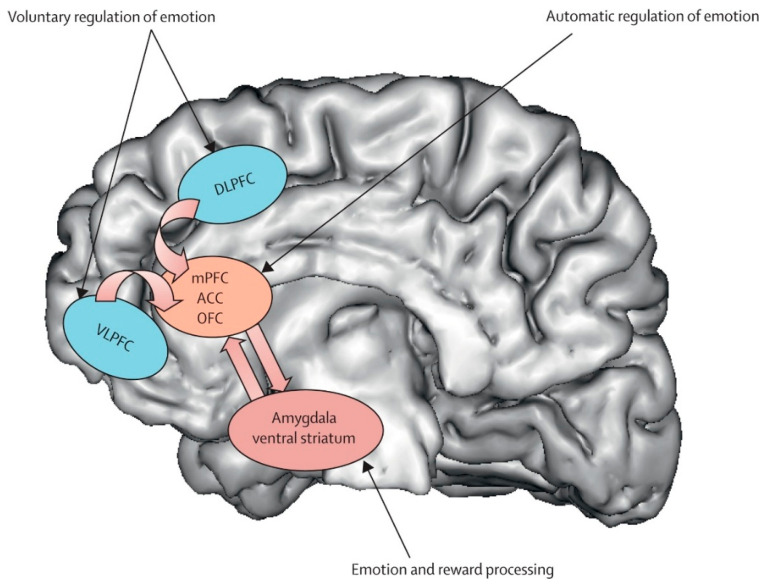
Major depressive disorder: new clinical, neurobiological, and treatment perspectives [61].

Prefrontal Cortex (DLPFC/VMPFC). The prefrontal cortex (PFC) represents the most consistently altered neural substrate in DS, with convergent evidence spanning over two decades of neurobiological research. The pattern of alterations specifically involves hypoactivation of the dorsolateral prefrontal cortex (DLPFC) and dysfunction of the ventromedial prefrontal cortex (VMPFC), creating functional compromises that correlate directly with depressive symptomatology. Meta-analytic evidence reveals widespread structural reductions in prefrontal regions in DS, with the left DLPFC showing 15–25% reductions in metabolic activity compared to healthy controls [53]. This hypoactivation becomes particularly evident during working memory, executive control, and cognitive emotion regulation tasks, manifesting through reduced cerebral perfusion and decreased glucose metabolism [54,55]. Functional magnetic resonance imaging studies have documented specific alterations in connectivity between the DLPFC and frontoparietal network, with 30–40% reductions in neural synchronization during complex cognitive tasks. These dysfunctions correlate significantly with neuropsychological assessments of executive functions, sustained attention, and working memory, providing a neurobiological foundation for the cognitive symptoms commonly observed in DS [56]. The VMPFC presents complementary alterations, characterized by anomalous hyperactivation during negative emotional stimulus processing and hypoactivation during voluntary emotional regulation tasks [57]. This pattern reflects a 35–50% compromise in top-down modulation effectiveness of limbic structures (Figure 2), determining reduced cognitive control over emotional responses [58]. The therapeutic implications of prefrontal dysfunction have been particularly well-demonstrated through repetitive transcranial magnetic stimulation (rTMS) research. High-frequency stimulation of the left DLPFC has shown robust antidepressant effects, with accelerated protocols achieving 60–75% remission rates in treatment-resistant patients [59]. The identification of specific connectivity profiles between stimulation sites and the subgenual anterior cingulate cortex has further refined targeting strategies, demonstrating how understanding neural correlates can directly inform therapeutic approaches [60].

**Figure 3:**
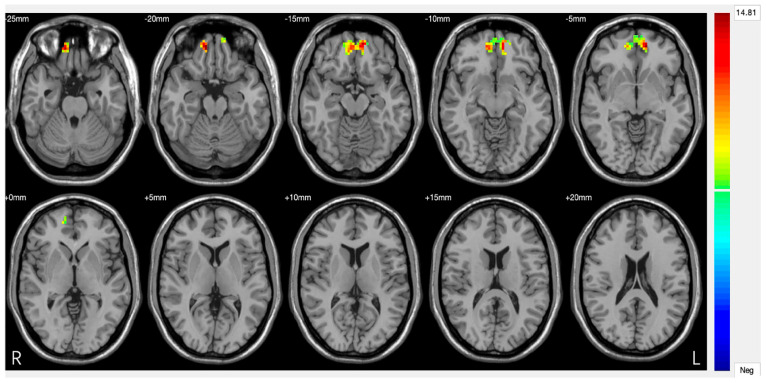
Significant differences in amygdala–prefrontal cortex (PFC) functional connectivity (FC) among the major depressive disorder (DS) with a history of suicide attempts (SA), DS without a history of SA, and HC groups. Significant at *p* < 0.001, corrected by Gaussian random field (GRF) correction [68].

Amygdala. The amygdala serves as a crucial neural hub in depressive pathophysiology, representing the primary centre for negative emotion processing and threat response coordination [62]. Functional alterations of this structure result in pathological amplification of negative emotional responses while compromising fear extinction mechanisms, significantly contributing to the emotional dysregulation characteristic of DS. Neuroimaging studies consistently document amygdala hyperactivation patterns in response to negative emotional stimuli, with BOLD activity increases of 40–60% compared to healthy controls [63]. This hyperresponsivity shows selective characteristics, being particularly pronounced for negatively valenced stimuli while paradoxically reduced for positive stimuli, suggesting specific dysregulation of emotional processing circuits rather than generalized emotional reactivity (Figure 3). The basolateral amygdala demonstrates specific alterations in synaptic plasticity, with reduced GABAergic inhibition effectiveness leading to increased neuronal excitability [64]. High-resolution magnetic resonance studies have revealed microstructural modifications, including 12–18% increases in cellular density that correlate with symptom severity [65]. Connectivity between the amygdala and VMPFC shows significant compromise in DS, with 30–45% reductions in functional synchronization during emotional regulation tasks [66]. This disconnection compromises prefrontal top-down modulation effectiveness, perpetuating amygdala hyperactivation and limiting cognitive emotional control capacity. Diffusion tensor imaging studies have identified corresponding white matter alterations in tracts connecting the amygdala to the orbitofrontal cortex, with 15–25% reductions in fractional anisotropy correlating with depressive symptom intensity [67].

Hippocampus. The hippocampus represents one of the brain structures most vulnerable to chronic stress and depression effects, showing progressive structural and functional alterations that correlate with depressive episode duration and recurrence [69]. This region’s central role in memory, learning, and hypothalamic–pituitary–adrenal axis regulation makes it particularly susceptible to the neuroplastic modifications that characterize DS pathophysiology. Structural neuroimaging meta-analyses have consistently documented 8–15% hippocampal volumetric reductions in DS patients, with more pronounced effects in anterior compared to posterior regions [70]. These reductions demonstrate a dose-dependent relationship with cumulative depressive episode duration, suggesting progressive neurodegenerative processes that accumulate over time with repeated episodes. Voxel-based morphometry analyses have identified specific gray matter density reductions in hippocampal CA1 and CA3 regions, areas particularly vulnerable to glucocorticoid neurotoxicity [71]. Post-mortem histological studies have confirmed these findings, demonstrating 12–20% reductions in pyramidal neuron number and compromised dendritic arborization associated with chronic glucocorticoid exposure and reduced neurotrophic factor availability [72]. Adult hippocampal neurogenesis shows significant compromise in DS, with 30–50% reductions in cell proliferation within the dentate gyrus. This neurogenesis deficit associates with reduced BDNF levels and altered neurotrophic signalling pathways, creating a self-perpetuating cycle where reduced neuroplasticity impairs the brain’s capacity for adaptive responses to stress and environmental challenges [73].

**Figure 4:**
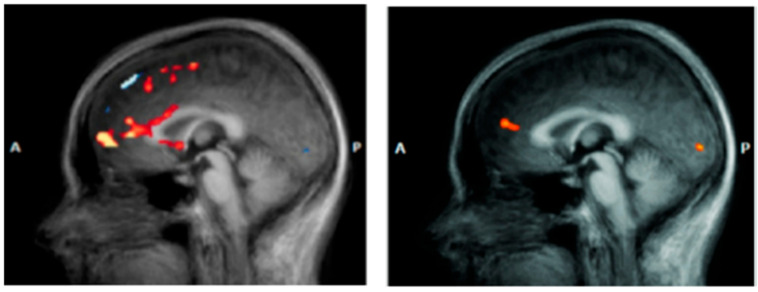
Anterior cingulate cortex (ACC) in a patient with DS (**left**) vs. a healthy subject (**right**) [77].

Anterior Cingulate Cortex (ACC). The anterior cingulate cortex (ACC) represents a crucial neural interface between cognition and emotion, playing fundamental integrative roles in conflict monitoring, attention regulation, and emotional response control [74]. In DS, the ACC demonstrates specific functional alterations with direct implications for both pathophysiological understanding and therapeutic response prediction (Figure 4). The ACC can be functionally subdivided into distinct regions that show different alteration patterns in depression. The subgenual portion (sgACC) demonstrates characteristic metabolic hyperactivation, with 20–40% increases in glucose uptake that correlate positively with depressive symptom severity [75]. This hyperactivation represents a robust biological marker of depressive state and has proven valuable for understanding treatment mechanisms and predicting therapeutic responses. The dorsal anterior cingulate cortex (dACC) presents a complementary hypoactivation pattern, particularly evident during tasks requiring cognitive control and conflict resolution [76]. Functional neuroimaging studies document 25–35% activation reductions during Stroop paradigms and executive control tasks, correlating with attentional deficits and executive function compromises characteristic of DS. The sgACC’s extensive anatomical connections with limbic structures, paralimbic regions, and brainstem nuclei involved in mood regulation undergo profound alterations in DS [10]. Anomalous functional connectivity increases between sgACC and amygdala contribute to negative emotional response amplification, while disrupted connectivity with prefrontal regions compromises cognitive control mechanisms (Table 1).

## 6. Comparative Neuroimaging Analysis Across Depressive Subtypes

Recent advances in neuroimaging technology and analytical methods have enabled detailed characterization of the distinct neurobiological signatures across depressive subtypes [78,79,80,81]. Multi-site fMRI studies involving over 2000 patients have identified robust biomarkers that distinguish between subtypes with 75–85% accuracy [82]. Key findings from comparative neuroimaging demonstrate that melancholic depression shows the most severe striatal hypoactivation and pronounced subgenual ACC hypermetabolism. Atypical depression demonstrates unique orbitofrontal hyperactivity and enhanced amygdala reactivity to social stimuli. Persistent depressive disorder exhibits chronic DMN dysfunction with progressive structural deterioration. Diffusion tensor imaging studies have revealed subtype-specific patterns of white matter integrity alterations [83]. Melancholic depression predominantly shows posterior white matter changes affecting the cingulum bundle and fornix. Atypical depression demonstrates anterior alterations in the corpus callosum and internal capsule. Chronic/PDD cases exhibit widespread white matter hyperintensities with progressive deterioration. Longitudinal neuroimaging studies tracking patients over 2–5 years have documented differential trajectories of brain structural changes across subtypes. Episodic DS shows partial recovery of hippocampal volume with effective treatment. Melancholic depression demonstrates persistent reward circuit alterations despite symptom remission. PDD exhibits progressive deterioration despite ongoing treatment [84].

## 7. Frontal Alpha Asymmetry on the EEG

Although the prefrontal cortex is central to research on the subject, the evolutionary aspect has recently been revisited. Indeed, one study demonstrated that frontal alpha asymmetry on the EEG is an important line of research linking early brain markers to the risk of affective disorders. It further specified that variation in the 5-HTTLPR genotype modulates frontal asymmetries on the resting-state EEG in children, suggesting a developmental mechanism underlying affective risk. This evidence is currently being explored [85].

## 8. Clinical Implications and Therapeutic Perspectives

The identification of specific neural correlates in DS has opened unprecedented opportunities for developing personalized diagnostic and therapeutic approaches. Understanding the neurobiological substrates of depression allows clinicians to move beyond symptom-based treatment selection toward biologically informed interventions that target specific dysfunctional circuits. Neurostimulation techniques, particularly repetitive transcranial magnetic stimulation and deep brain stimulation, exemplify how neurobiological knowledge translates into therapeutic innovation [37]. The success of left DLPFC stimulation in treating depression directly reflects our understanding of prefrontal hypoactivation in the disorder, while emerging techniques targeting specific network connections promise even greater precision in therapeutic interventions. Pharmacological approaches are similarly benefiting from neurobiological insights, with drug development increasingly focused on specific circuit dysfunctions rather than broad neurotransmitter effects. Novel approaches targeting neuroinflammation, neuroplasticity enhancement, and network connectivity modulation represent promising avenues for individuals who do not respond to traditional monoaminergic interventions. The integration of neuroimaging biomarkers into clinical practice holds promise for improving treatment selection and monitoring therapeutic response [86]. Current research focuses on machine learning classification, with advanced algorithms achieving 80–90% accuracy in subtype classification using multimodal neuroimaging data [87]. Blood-based biomarkers (Table 2) integrate neuroimaging findings with peripheral markers of inflammation, neuroplasticity, and HPA axis function [88]. Digital biomarkers utilize smartphone-based ecological momentary assessment combined with wearable device data to capture real-world functional correlates of neurobiological subtypes. Functional connectivity patterns, structural alterations, and metabolic signatures may eventually guide personalized treatment decisions, moving psychiatric practice toward the precision medicine approaches already transforming other medical specialties [89]. Modern models of environmental sensitivity/differential susceptibility are increasingly used and posit that some individuals are more sensitive to both adverse and favorable environments. This perspective differs from traditional depression risk models and could be an additional analytical element to consider when assessing the subjective depressive profile. In the clinical field, the analysis of biomarkers and neuroimaging allows us to better understand the pathologies of the depressive spectrum; however, it must also be considered that in low- and middle-income countries, this research is almost non-existent, due to strong limitations in cost/accessibility, preventing its structured and methodical use.

## 9. Conclusions

The investigation of neural correlates across depressive subtypes is unveiling a remarkably complex picture of distinct neurobiological signatures that extend beyond the traditional “one-size-fits-all” conceptualization of DS. Converging evidence points to specific patterns of dysfunction involving different circuits across subtypes: reward processing in melancholic depression, social–emotional circuits in atypical depression, and progressive structural deterioration in persistent depressive disorder. Perhaps even more significantly, research has demonstrated that different depressive subtypes cannot be understood simply as variations of a single disorder, but rather as distinct neurobiological entities characterized by unique patterns of circuit dysfunction, treatment response, and clinical trajectory. The identification of subtype-specific neurobiological signatures offers genuine cause for optimism in advancing precision psychiatry approaches. The translation of these findings into biomarker-based treatment selection algorithms is already producing tangible benefits, with neuroimaging-guided interventions showing superior outcomes compared to traditional trial-and-error approaches. Even more compelling, the framework provided by subtype-specific neurobiology is guiding the development of novel interventions that can be precisely targeted to individual patterns of dysfunction. However, critical challenges remain in translating these research findings into routine clinical practice. The heterogeneity within subtypes requires more sophisticated approaches to biological stratification, while the integration of multiple assessment modalities—neuroimaging, peripheral biomarkers, and digital phenotyping—demands substantial infrastructure development and cost considerations. Future research should prioritize the development of clinically feasible biomarker panels that can be implemented in routine psychiatric practice. The emerging understanding of gene-environment interactions, epigenetic mechanisms, and developmental trajectories highlights the need for more comprehensive models that integrate neurobiological findings with broader biopsychosocial factors. Perhaps most crucially, future research should maintain its focus on translational applications that directly benefit patients. While scientific understanding of depression subtype neurobiology has advanced substantially, the ultimate test of this knowledge lies in its capacity to reduce suffering and improve outcomes for individuals experiencing different forms of depression. This requires continued collaboration between basic researchers, clinicians, and patients to ensure that scientific advances are effectively translated into meaningful improvements in care. The field currently finds itself at a critical juncture where decades of fundamental research are beginning to yield practical applications that can transform treatment approaches. The neural correlates of depressive subtypes are today driving the development of precision interventions that offer hope for more effective, personalized, and accessible treatments. As our understanding continues to evolve, the prospect of truly biomarker-guided therapeutic approaches for treating different forms of depression becomes increasingly realistic, promising a future where treatment selection is guided not by symptom description alone but by a detailed understanding of individual neurobiological profiles and their specific therapeutic needs.

## Figures and Tables

**Table 1:** Neuroanatomical and functional differences between a healthy subject and a subject with DS. Source: Authors.

Neuroanatomical Areas	Healthy Subject	Major Depression	Melancholic Depression	Atypical Depression	Persistent Depression
Dorsolateral Prefrontal Cortex (DLPFC)	Normal metabolic activity and functional connectivity with frontoparietal networks. Essential for executive functions and working memory.	15–25% reduction in metabolic activity. 30–40% reduction in functional connectivity with the frontoparietal network.	Severe hypoactivation (35–45% reduction) particularly during reward processing. Enhanced response to high-frequency rTMS.	Moderate hypoactivation (20–30% reduction) with preserved capacity for mood reactivity.	Progressive hypoactivation correlated with illness duration. Reduced neuroplastic response capacity.
Ventromedial Prefrontal Cortex (VMPFC)	Crucial for emotional regulation and top-down limbic modulation. Balanced connecti-vity with ACC and limbic regions.	Anomalous hyperactivation during negative processing. 35–50% compromise in limbic modulation effectiveness.	Severe dysfunction in reward valuation. Altered connectivity with ventral striatum (45–60% reduction).	Hyperreactivity to social–emotional stimuli. Preserved emotional reactivity but altered social reward processing.	Chronic dysfunction with progressive structural alterations. Reduced emotional regulation capacity.
Amygdala	Balanced responsivity to emotional stimuli with effective prefrontal modulation. Average volume ≈1.2 cm^3^ per hemisphere.	40–60% BOLD activity increases to negative stimuli. 30–45% reduction in prefrontal connectivity.	Moderate hyperactivation with specific deficits in reward-related processing. Enhanced stress reactivity.	Pronounced hyperreactivity to interpersonal rejection cues (50–70% increase). Enhanced social threat detection.	Chronic hyperactivation with structural changes. 15–25% volume increases with cellular density alterations.
Hippocampus	Critical for memory consolidation and HPA axis regulation. Normal volumetric integrity and neuroplastic capacity.	8–15% volumetric reductions, more pronounced in anterior regions. Progressive volume loss with episode recurrence.	Severe volume reductions (15–25%) correlated with HPA axis dysfunction. Marked neurogenesis impairment in the dentate gyrus.	Moderate volume reductions (8–12%) with preserved neurogenesis capacity. Less pronounced structural alterations.	Progressive volume loss (12–20%) correlated with illness duration. Chronic neurogenesis suppression and BDNF deficits.
Anterior Cingulate Cortex (ACC)	Integrates cognitive and emotional	Subgenual ACC: 20–40% metabolic	Pronounced subgenual hypermetabolism	Moderate subgenual activation with	Chronic dysfunction with progressive

**Table 2:** Biomarker-based treatment response patterns.

Depressive Subtype	Neurobiological Biomarkers	First-Line Treatments	Response Rates	Treatment Duration	Neuroimaging Predictors
Major Depression	Moderate DLPFC hypoactivation, DMN hyperconnectivity, balanced limbic dysfunction	SSRIs, SNRIs, CBT, rTMS (left DLPFC)	60–75% response rate	6–12 weeks acute treatment	DLPFC connectivity strength, sgACC metabolism
Melancholic Depression	Severe reward circuit dysfunction, HPA axis hyperactivity, pronounced sgACC hypermetabolism	TCAs, ECT, dopaminergic augmentation, high-frequency rTMS	70–85% with ECT, 45–60% with medications	8–16 weeks acute treatment	Striatal activation, cortisol levels, sgACC hypermetabolism
Atypical Depression	Enhanced limbic social reactivity, orbitofrontal hypermetabolism, and preserved reward sensitivity	MAOIs, SSRIs, behavioral activation, interpersonal therapy	65–80% response rate	8–12 weeks acute treatment	Amygdala social reactivity, orbitofrontal metabolism
Persistent Depressive Disorder	Chronic DMN dysfunction, progressive structural changes, reduced neuroplasticity	Combined psychotherapy–pharmacotherapy, maintenance treatment, and neuroplasticity enhancers	40–60% response rate	12–24+ weeks treatment	White matter integrity, hippocampal volume, and BDNF levels

## Data Availability

Not applicable as no new data were generated in this review article.
